# The role of systemic ımmune ınflammatory ındex in showing active lesion ın patients with multiple sclerosis: SII and other inflamatuar biomarker in radiological active multiple sclerosis patients

**DOI:** 10.1186/s12883-023-03101-0

**Published:** 2023-02-10

**Authors:** Seyda Figul Gokce, Asli Bolayır, Burhanettin Cigdem, Bulent Yildiz

**Affiliations:** 1 School of Medicine, Neurology Department, Sivas Cumhuriyet University, Sivas, Turkey; 2 School of Medicine, Radiology Department, Sivas Cumhuriyet University, Sivas, Turkey

## Abstract

**Background:**

Multiple sclerosis (MS) has two pathophysiological processes, one inflammatory and the other degenerative. We investigated the relationship between active lesions on magnetic resonance imaging showing the inflammatory phase in MS patients and serum parameters that can be used as inflammatory biomarkers. Thus, we aim to detect the inflammatory period in clinical and radiological follow-up and to reveal the period in which disease-modifying treatments are effective with serum parameters.

**Methods:**

One hundred eighty-six MS patients presented to our hospital between January 2016 and November 2021 and 94 age- and sex-matched healthy volunteers were recruited for our study. While 99 patients had active lesions on magnetic resonance imaging, 87 patients did not have any active lesions. Neutrophil/lymphocyte ratio (NLR), platelet/lymphocyte ratio (PLR), and monocyte/lymphocyte ratio (MLR) were determined. The SII (systemic immune inflammatory index) value was calculated according to the platelet X neutrophil/lymphocyte ratio formula.

**Results:**

NLR, MLR, PLR and SII values were found to be statistically significantly higher in MS patients than in the control group. The NLR, MLR, PLR and SII were higher in the active group with gadolonium than in the group without active lesions. In addition, the cutoff values that we can use to determine the presence of active lesions were 1.53, 0.18, 117.15, and 434.45 for NLR, MLR PLR and SII, respectively.

**Conclusions:**

We found that all parameters correlated with radiological activity. In addition, we showed that we can detect the inflammatory period with high sensitivity and specificity with the cutoff value used for SII and PLR. Among these easily accessible and inexpensive evaluations, we concluded that SII, including the values in the PLR formula, can come to the fore.

## Introduction

Multiple sclerosis (MS), a chronic inflammatory demyelinating disease, is a disorder of the central nervous system (CNS) that most commonly causes disability in young adults after trauma [1, 2]. Genetic and environmental risk factors are placed in its multifactorial etiology. It has multiple subtypes, and the most common of these is relapsing-remitting MS (RRMS). It accounts for approximately 85% of cases. RRMS is characterized by recurrent neurological symptoms lasting from a few days to weeks. These symptoms show partial or almost complete improvement with spontaneous or some treatment options, such as systemic steroid use. MRI reflections of relapses may be seen in the development of a focal area in the magnetic resonance imaging (MRI) T1 sequence with contrast enhancement after gadolinium injection (Gd+) and/or hyperintense new lesion development or volume expansion in the T2 sequence [3]. Inflammation is thought to be dominant at this active stage of the disease, which is the period when current disease modified treatments (DMT) in MS are more effective [4]. Within approximately 10-15 years from disease onset, if the patients do not receive effective treatment, most patients with RRMS develop secondary progressive MS (SPMS), characterized by gradual clinical deterioration that does not respond adequately to any available therapy. SPMS can be said to involve a chronic degenerative process with or without relapses, in which the inflammatory process plays a lesser role in the central nervous system (CNS) and increases disability in patients.

CNS inflammation and neurodegeneration, which occur with activation of innate and acquired immune cells triggered by autoimmunity, play a role in the pathogenesis of MS [5, 6]. These changes result in several changes, such as demyelinating lesions consistent with myelin damage in the white and gray matter and atrophy, indicating axonal loss in the brain and spinal cord [7]. In addition to the clinic, radiological evaluation with MRI is extremely important in the diagnosis of MS disease and plays a key role in the follow-up of the disease. Identifying biomarkers that will help evaluate the disease process and treatment efficacy is known to provide patients with a versatile and more sensitive follow-up. Light chain neurofilaments are a marker that gives strength to the clinicians in monitoring used for this purpose [8]. However, its use in practice has several limitations, so researchers investigate easily accessible, reliable and cost-effective indicators of its use that will allow routine utilization.

The differential count of white blood cells is a widely used biomarker to indicate systemic inflammation. Many ratios, such as neutrophil/lymphocyte (NLR), platelet/lymphocyte (PLR), and monocyte/lymphocyte (MLR) ratios, obtained by dividing the cell counts by each other, which are thought to be superior to the use of white blood cell subtypes alone in demonstrating inflammation, are currently in practice [9]. For example, cerebrovascular diseases, cancers, and autoimmune diseases have been the subject of studies on this subject [10–12]. MS was also involved in these studies. It is known that the NLR value increases in early MS [13]. In another study by Hemond et al., high NLR and MLR levels were found to be associated with MS-related disability, independent of all demographic, clinical, treatment-related, and psychosocial variables [14]. In a study comparing PLR with NLR, a higher value was found in aquaporin-4-antibody-positive neuromyelitis optica spectrum disorders (NMOSDs) than in MS [15].

The systemic immune-inflammatory index (SII), which is calculated according to the peripheral platelet count X neutrophil count/lymphocyte count formula, has been used to determine the prognosis in many cancer types, especially hepatocellular cancer and other diseases that progress with changes in inflammatory backgrounds [16, 17]. However, no study in the literature has investigated the relationship between SII levels and the pathophysiology of inflammation in MS.

In this study, NLR, PLR, MLR and SII levels were planned to be evaluated in both patient groups with active contrast-enhancing lesions and without contrast enhancing lesions groups on MRI and in healthy controls. In this way, we investigated whether the inflammatory process in which DMT drugs are effective (with the idea that gadolonium involvement in MRI reflects inflammation) can be detected in the blood through NLR, PLR, MLR and SII values as well as whether it can be used as a biomarker in terms of prognosis and disability.

There have been some concerns about the use of gadolonium recently. In particular, the accumulation of this substance in the brain and its neurodegenerative consequences have been discussed [18]. In addition, we aimed to examine the preferability of NLR, PLR, MLR and SII levels as an alternative to gadolonium use in subgroups of patients with and without active lesions on MRI.

## Methods

### Determination of the study group

In our study, patient records from January 2016 to November 2021 were reviewed retrospectively. One hundred eighty-six patients who presented to our hospital and who were diagnosed with relapsing remitting MS by two neurologists and 94 age- and sex-matched volunteers who gave blood for other reasons were recruited for our study. Evaluation of the data was performed in 2021. The 2010 McDonald's criteria were used to diagnose MS. The McDonald Criteria 2010 was revised at the end of 2017 [19], but since we wanted to include 2016 and 2017’s patients in our study too, the McDonald 2010 criteria were used in the joint diagnosis of patients until 2021. In this study, we aimed to identify new neurological examination findings described by the patient or detected independently by two neurologists after exclusion of causes such as fever and infection lasting more than 24 hours and detect any accompanying gadolonium-enhancing T1 lesions in the brain or spinal cord radiologically consistent or inconsistent with clinical findings. In addition, an active patient group was formed with the detection of gadolonium-enhancing T1 lesions in the brain or spinal cord at the time of diagnosis and in the periodic follow-ups of patients with or without new clinical complaints or new neurological examination findings. The noncontrast patient group consisted of patients who did not have brain or spinal cord lesions detected with active gadolonium in their periodic follow-ups, did not have any new complaints or examination findings in the last three months, and did not receive acute attack treatment.

Patients with contrast-enhancing lesions after gadolinium injection (Gd+) were included in the patient group with active lesions. Patients who did not have any Gd+ lesions were included in the patient group without active lesions. While the number of patients with active lesions in the patient group was 99, the number of patients without active lesions was 87. The EDSS value (Expanded Disability Status Scale) was used to evaluate the disability of the patients. Disease duration was calculated on the basis of the time of initial diagnosis of the patient, and the treatments received by the patients were those they were receiving at the time of blood collection. Disease-modifying therapies act with different mechanisms of action. For example, while interferons may cause lymphopenia, lymphocytosis may occur with natalizumab, which controls lymphocyte passage to the central nervous system [20, 21].

Therefore, we included patients at the time of initial diagnosis, patients who did not receive treatment, patients who took medication not affecting lymphocyte, neutrophil, monocyte and platelet counts and those with no history of immunosuppressant treatment.

The rights of all participants were protected according to the Declaration of Helsinki. Patients under 18 years of age, patients with active infection, diabetes mellitus, hypertension, acute/chronic liver or kidney failure, active or chronic inflammatory disease (such as inflammatory bowel disease, Sjögren's syndrome), other autoimmune disease, pregnant patients, patients with existing malignancy, and patients with a history of surgical intervention, acute myocardial infarction or trauma over the last 3 months were not included in our study.

The control group was randomly generated from 94 age- and gender-matched people who applied to our outpatient clinic between 2016 and 2021, did not have any complaints or systemic diseases and were asked to document that they were healthy.

Ethics committee approval of our study was obtained from Sivas Cumhuriyet University Non-Interventional Ethics Committee on 21.10.2020 under number 2020-10/23.

### Evaluation of biochemical and hematological parameters

Dry tubes were used for biochemical analysis, and EDTA tubes were used for hematological tests. Complete blood count analysis of blood samples taken from the left antecubital vein of the subjects at rest was performed using Diagon kits on the Mindray BC-6800 device, and kits from the same company were used with the fully automatic nephelometric method on the Beckman Coulter AU5800 (Beckman Coulter Inc, Hialeah, Florida) device for biochemical analyses. In our clinic, if any imaging containing gadolonium is to be performed, we have a practice in which kidney function tests are requested on the same day. In addition to this test in MS patients, complete blood count, sedimentation, CRP, Vitamin D, folic acid, Vitamin B12, and thyroid function tests were added. Therefore, blood samples taken within 24 hours were included in the study without storage and were evaluated within the same day.

NLR, PLR and MLR values were obtained by dividing the cell counts in the measured complete blood count by each other. The SII value was calculated according to the platelet X neutrophil/lymphocyte formula.

### Evaluation with cranial magnetic resonance imaging

Cranial images obtained by the Department of Radiology of Sivas Cumhuriyet University on Siemens brand Magnetom Aera 2013 Model 1.5 Tesla magnetic resonance imaging device were evaluated with the automation system over the Sectra Uniview system. Demyelinating lesions with contrast enhancement after gadolinium injection (Gd +) in the T1 examination of these MR images in the axial, coronal and sagittal planes were defined as active lesions. The presence of active lesions was evaluated by an independent radiologist and neurologist, and patients were included in the patient group with active lesions accordingly. The other patient group without active lesions consisted of patients who did not have Gd + lesions. Additionally, the patients with active lesions were divided into 3 groups by the number of lesions they had (1–5, 6–10 and 11 and above).

### Statistical method

NCSS (Number Cruncher Statistical System) 2007 (Kaysville, Utah, USA) was employed for statistical analyses. The conformity of the quantitative data to the normal distribution was tested with the Shapiro‒Wilk test and graphical examinations. Whether the distribution of continuous data was close to normal was examined with the Kolmogorov‒Smirnov test. When continuous data showed a normal distribution, they were expressed as the mean ± standard deviation (SD); otherwise, they were expressed as the median and minimum-maximum, while categorical data were expressed as frequencies and percentages. During the comparison of quantitative data, Student’s t test was used to compare two groups showing a normal distribution, and the Mann‒Whitney U test was used to compare two groups not showing a normal distribution. One-way analysis of variance and binary evaluations with Bonferroni correction were used to make more than two intergroup comparisons of normally distributed quantitative variables. The Kruskal‒Wallis test and Dunn-Bonferroni test were used for comparisons between groups of more than two nonnormally distributed quantitative variables. Fisher-Freeman-Halton exact test was used to compare qualitative data. Spearman correlation analysis was employed to evaluate the relationships between quantitative variables. Diagnostic screening tests (sensitivity, specificity, PKD, NKD) and ROC curve analysis were used to determine the cutoff. Statistical significance was accepted as *p*<0.05.

The sample size and power analysis of the study were made with the G*Power 3.1 test program. The sample size was determined by reference to the studies of Demirci et al. Assuming ∝=0.05, effect size d=0.65, 87 MS patient groups and 47 control groups were calculated for 0.95 (1-β) power. However, since our study was designed retrospectively, it was conducted with 186 MS patients and 94 control groups. The power of our study was calculated as 0.99 in Post Hoc analysis [22, 23].

## Results

The research was conducted between January 2016 and November 2021 in Sivas Cumhuriyet University Hospital, Department of Neurology, with a total of 280 cases, 73.6% (*n*=206) female and 26.4% (*n*=74) male. The group of patients was divided into two subgroups: patients with and without active lesions. In the group of patients with active lesions (*n*=99), 73.7% (*n*=73) were females, while in the group without active lesions (*n*=87), 78.2% (*n*=68) were females. This rate was 69.1% (*n*=65) in the patients in the control group. There was no difference in sex among the three groups (*p*=0.392). The mean age was 33.85±9.6 years in the patient group with active lesions (*n*=99) and 32.72±7.4 years in the group without active lesions. The mean age was 33.44±8.0 years in the patients in the control group. There was no difference among the three groups in terms of sex or age (*p*=0.656). When the number of lesions in the patient group with active lesions was analyzed, the number of lesions was between 1-5 in 54.5% (*n*=54), between 6-10 in 16.2% (*n*=16), and between 11 and above in 29.3% (*n*=29). When the localizations of these lesions were analyzed, it was determined that 77.8% (*n*=77) were supratentorial, 17.2% (*n*=17) were infratentorial, and 5.1% (*n*=5) were spinal. The disease period in this patient group changed between 0 and 22 years, and the mean disease period was 5.05±4.65 years. EDSS measurements changed between 0 and 6 values, and the mean was 1.11±0.49. %54.3 (*n*=101) of the patient group was under treatment during this study. In terms of laboratory parameters, the patient group had high monocyte, platelet, and neutrophil counts, while the control group had a statistically significantly higher lymphocyte count (*p*=0.001). When the patient group was divided into 2 groups according to the presence of active lesions, the number of monocytes, platelets and neutrophils in the group with active lesions was found to be statistically significantly higher than that in both the patient group without active lesions and the control group (*p*=0.001, *p*=0.001, *p*=0.001, respectively). Additionally, the number of lymphocytes in the group with active lesions was found to be significantly lower than that in both the patient group without active lesions and the control group (*p*=0.001). There was no statistically significant difference between the patient and control groups in terms of CRP measurements and eosinophil counts (*p*=0.425, *p*=0.104). When the MLR, PLR, NLR and SII ratios were examined, all 4 ratios were found to be statistically significantly higher in the patient group versus the control group (*p*=0.001). Similarly, MLR, NLR, PLR and SII values ​​in patients with active lesions were statistically significantly higher than in patients without active lesions, according to the analysis of the subgroup in the patient group (respectively *p*=0.001, *p*=0.001, *p*=0.001, *p*=0.001) (Table 1). In addition, when the patient group was divided into 2 subgroups according to the status of receiving treatment, the PLR ​​values ​​in the group receiving treatment were statistically significantly lower than those in the group not receiving treatment (*p*=0.042), but there was no difference in terms of the MLR, NLR and SII values between the two groups.

**Table 1:** The comprassion of the basal demographic and laboratory findings and MLR, PLR, NLR, SII values of patient and control groups

	**Patient Group**			**Control Group(*n* = 94)**	***p***
	**Total(*n* = 186)**	**Active Lesion( +)(*n* = 99)**	**Active Lesion(-)(*n* = 87)**		
**Age(year) (mean ± SD)**	33.21 ± 7.6	33.85 ± 9.6	32.72 ± 7.38	33.44 ± 8.04	^b^0.656
**Gender(female(n,%))**	141(75.8%)	73 (73.7%)	68 (78.2%)	65 (69.1)	^c^0.392
**Disease Duration(year)(mean ± SD)**	5.05 ± 4.65	**-**	-	-	^**−**^
**EDSS(mean ± SD)**	1.11 ± 0.49	**-**	-	-	^**−**^
**Lesion count**		**1–5:** 54 (54.5%) **6–10:** 16(16.2%) ** ≥ 11:** 29 (29,3%)			
**Lesion localisation**	-	**Supratentorial:**77(77.8%) **Infratentorial:17**(17.2%) **Spinal:**5(5.1%)	-	-	^**−**^
**CRP(mg/dL)** **(median(min–max))**	2.3 (0.1–9.8)	2.1 (0.1–9.8)	2.4 (0.1–8.5)	2.4(1–7.1)	^**b**^**0.425**
**Monocyte count(10**^**9**^**/L) (mean ± SD)**	0.42 ± 0.19	0.50 ± 0.23	0.42 ± 0.17	0.34 ± 0.10	^**b**^ 0.001**
**Lymphocyte count(10**^**9**^**/L) (median(min–max))**	2.2 (0.9–4)	1.8 (0.9–2.9)	2.2 (0.9–3.5)	2.6 (1.4–4)	^**a**^ 0.001**
**Platelet count(10**^**9**^**/L)** **(mean ± SD)**	281.11 ± 69.06	330.72 ± 63.74	285.92 ± 54.99	224.41 ± 36.62	^**b**^0.001**
**Eosinophil count(10**^**9**^**/L) (median(min–max))**	0.1 (0–0.4)	0.1 (0–0.4)	0.1 (0–0.4)	0.1 (0–0.2)	^a^0.104
**Neutrophil count(10**^**9**^**/L) (median(min–max))**	3.6 (1–14)	4.2 (2.1–14)	3.6 (1–14)	3.1 (1–4.9)	^**a**^ 0.001**
**MLR(median(min–max))**	0.2 (0–1.3)	0.3 (0.1–1.3)	0.2 (0–0.7)	0.1 (0–0.3)	^**a**^0.001**
**PLR(median(min–max))**	122.8 (43.2–421.8)	189.8 (83.8–421.8)	135 (61.3–351.3)	89.3 (43.2–189)	^**a**^0.001**
**NLR(median(min–max))**	1.5 (0.4–10.6)	2.5 (1–10.6)	1.5 (0.5–10.6)	1.2 (0.4–2.8)	^**a**^0.001**
**SII(median(min–max))**	452.7 (71.1–3215.6)	797.9 (273–3215.6)	478.1 (151.9–3215.6)	234.2 (71.1–674)	^**a**^0.001**

*EDSS* Expanded Disability Status Scale, *CRP* C- Reactive protein, *MLR* Monocyte/ lymphocyte ratio, PLR Platelet/ lymphocyte ratio, *NLR* Neutrophil / lymphocyte ratio, *SII* Systemic immune-inflammatory index

^*a*^*Kruskal Wallis Test; *^*b*^*One Way Anova Test; *^*C*^*Fisher Freeman Halton Test* All values are presented mean ± standard deviation(SD) or median value (min–max); **p<0.05

**Fig. 1:**
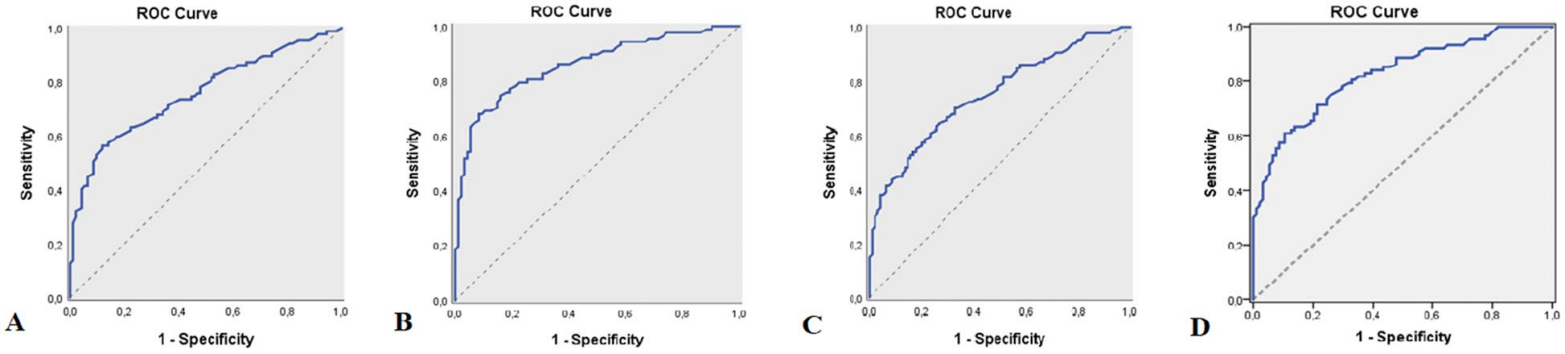
**A** ROC curve for MLR measurement by MS presence(AUC: 0.758), **B**: ROC curve for PLR measurement by MS presence(AUC:0.858), **C**: ROC curve for NLR measurement by MS presence(AUC:0.751), **D**: ROC curve for SII measurement by MS presence(AUC:0.826)

**Fig. 2:**
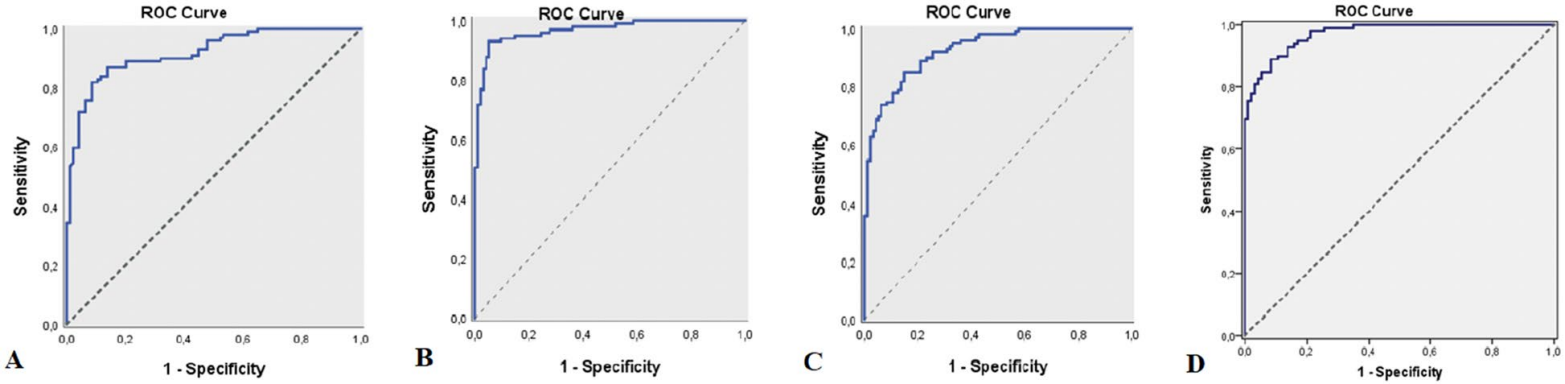
**A** ROC curve for MLR measurement by active lesions presence(AUC: 0.919), **B**: ROC curve for PLR measurement by active lesions presence(AUC:0.967), **C**: ROC curve for NLR measurement by active lesions presence(AUC:0.927), **D**: ROC curve for SII measurement by active lesions presence (AUC:0.972)

Based on the significance of the MLR, PLR, NLR and SII ratios, ROC analysis and diagnostic screening tests were used to calculate the cutoff point for the MLR, PLR, NLR and SII ratios to define MS. According to the presence of MS, the cutoff value for MLR was 0.17 (sensitivity: 55.17%; specificity: 88.30%; positive predictive value: 81.40; negative predictive value: 68.00; AUC: 75.8%, SE: 3.6%), the cutoff value for PLR was 115.59 (sensitivity: 66.67%; specificity: 91.49%; positive predictive value: 87.90; negative predictive value: 74.80; AUC: 85.8%; SE: 2.8%), the cutoff value for NLR was 1.36 (sensitivity: 63.22%; specificity: 74.47%; positive predictive value: 69.60; negative predictive value: 68.60; AUC: 

45.1%; SE: 3.6%), and the cutoff value for SII was 412.53 (sensitivity: 60.62%; specificity: 89.36%; positive predictive value: 84.10; negative predictive value: 71.20; AUC: 82.6%, SE: 3%) (Table 2, Fig. 1A). Furthermore, based on the elevation of MLR, PLR, NLR and SII values in patients with active lesions, ROC analysis and diagnostic screening tests were used to calculate the cutoff point for MLR, PLR, NLR and SII ratios to define active lesions in MS patients. According to the presence of active lesions in MS patients, the cutoff value for MLR was 0.18 (sensitivity: 82.83%; specificity: 91.49%; positive predictive value: 91.00; negative predictive value: 82.70; AUC: 91.9%, SE: 1.9%), the cutoff value for PLR was 117.15 (sensitivity: 91.92%; specificity: 94.68%; positive predictive value: 94.80; negative predictive value: 91.80; AUC: 96.7%; SE: 1.2%), the cutoff value for NLR was 1.53 (sensitivity: 83.84%; specificity: 85.11%; positive predictive value: 85.60; negative predictive value: 83.30; AUC: 92.7%; SE: 1.7%), and the cutoff value for SII was 434.45 (sensitivity: 88.89%; specificity: 91.49%; positive predictive value: 91.70; negative predictive value: 88.70; AUC: 97.2%, SE: 0.9%) (Table 3, Figure 2A, B, C, D).

**Table 2:** Diagnostic Screening Tests by presence of MS and ROC Curve Results for MLR, PLR, NLR, SII

	**Diagnostic Scan**					**ROC Curve**		***p***
	**Cut off**	**Sensitivite**	**Spesifisite**	**Positive Predictive Value**	**Negative** **Predictive Value**	**Area**	**95% Confidence Interval**	
**MLR**	** ≥ *0.17***	55.17	88.30	81.40	68.00	**0.758**	0.687–0.829	0.001**
**PLR**	** ≥ *115.59***	66.67	91.49	87.90	74.80	**0.858**	0.803–0.913	0.001**
**NLR**	** ≥ *1.36***	63.22	74.47	69.60	68.60	**0.751**	0.680–0.821	0.001**
**SII**	** ≥ *412.53***	60.92	89.36	84.10	71.20	**0.826**	0.766–0.885	0.001**

*MLR* Monocyte/ lymphocyte ratio, *PLR* Platelet/ lymphocyte ratio, *NLR* Neutrophil / lymphocyte ratio, *SII* Systemic immune-inflammatory index

**p<0.05

**Table 3:** Diagnostic Screening Tests by presence of active lesions in MS patients and ROC Curve Results for MLR, PLR, NLR, SII

	**Diagnostic Scan**					**ROC Curve**		***p***
	**Cut off**	**Sensitivite**	**Spesifisite**	**Positive Predictive Value**	**Negative** **Predictive Value**	**Area**	**95% Confidence Interval**	
**MLR**	** ≥ *0.183***	81.82	91.49	91.00	82.70	**0.919**	0.881–0.957	0.001**
**PLR**	** ≥ *117.15***	91.92	94.68	94.80	91.80	**0.967**	0.945–0.990	0.001**
**NLR**	** ≥ *1.53***	83.84	85.11	85.60	83.30	**0.927**	0.893–0.961	0.001**
**SII**	** ≥ *434.45***	88.89	91.49	91.7	88.7	**0.972**	0.954–0.989	0.001**

*MLR* monocyte/ lymphocyte ratio, *PLR* platelet/ lymphocyte ratio, *NLR* neutrophil / lymphocyte ratio, *SII* Systemic immune-inflammatory index

**p<0.05

In the patient group, there was a weak positive and statistically significant relationship between EDSS score and PLR measurements (*r*=0.160; *p*=0.029; *p*<0.05). However, there was no statistically significant relationship between EDSS score and MLR, NLR and SII measurements (*p*=0.484, *p*=0.367, *p*=0.080, respectively). Additionally, there was a weak positive and statistically significant relationship between disease duration and PLR measurements in the patient group (*r*=0.250; *p*=0.013; *p*<0.05). However, there was no statistically significant relationship between disease duration and MLR, NLR or SII measurements (*p*=0.683, *p*=0.389, *p*=0.432, respectively). Similarly, no statistically significant correlation was found between lesion localization or lesion number and MLR, PLR, NLR, and SII measurements in the patient group (*p*=0.556, *p*=0.813, *p*=0.976, *p*=0.997, *p*=0.117, *p*=0.577, *p*=0.816, *p*=0.517, respectively) .

## Discussion

The MLR, PLR, NLR, and SII rates were high compared to the healthy controls in the patient groups with or without active lesions on MRI in our study. Additionally, these rates were found to be significantly higher in the patient group with an active lesion compared to the group without active lesion. The MLR, PLR, NLR, and SII rates have higher specificity and sensitivity in detecting the active lesion rather than diagnosing the disease based on the results of our study. Additionally, we demonstrated that values above 0.17 for MLR, 115.59 for PLR, 1.36 for NLR, and 412.53 for SII can be used with moderate specificity and sensitivity in detecting MS and that cutoff values above 0.18 for MLR, 117.15 for PLR, 1.53 for NLR, and 434.45 for SII can be beneficial as biomarkers with high specificity and sensitivity in detecting radiological activity. When these values are evaluated individually, the rates with both the highest specificity and sensitivity and the largest area under the curve are SII and PLR. In this respect, these two values seem to be superior to other rates in detecting active lesions and the active process of the disease in MS patients.

The relationship between clinical activity and MRI findings in MS and inflammatory biomarkers was first investigated a long time ago. The evaluation in which clinical and radiological correlations were determined with serum nitric oxide metabolites, nitrate and nitrite levels is a good example of clinical activity and MRI findings .In this study, the correlation of these increased metabolites with the benign disease process was emphasized [24].

Later studies of blood parameter ratios, especially with NLR, was found in many diseases and MS. In a study by Bisgaard et al., NLR was found to be higher in MS patients with relapses than in those without [25]. Similarly, in our study, this rate was found to be high in patients with gadolonium-enhancing lesions. In another evaluation, the NLR rate was found to be high in patients who relapsed at the onset of RRMS [26].

The results of Demirci et al.'s study are also similar. In their study, they found a higher rate of NLR in patients in the relapse period than in the remission period. They also identified NLR as an independent predictor of disease progression. In this study, no evaluation was made on whether the patients received treatment [22]. Yetkin et al. examined MS patients who did not receive treatment and found a relationship between higher NLR values and the need for more effective treatment in the relapsed MS patient group. Similar to our study, Yetkin et al. found no relationship between disability and NLR [27].

In our study, a relationship was found between the increase in MLR and patients with active lesions. Examining brain atrophy and T2 lesions that we did not evaluate in our study, Hemond et al. found a correlation between a high MLR and brain atrophy but did not find a relationship with T2 lesions [14]. Another study showed that, in addition to NLR, a high MLR was correlated with a significant increase in the risk of relapse over a 2-year period in MS patients [28].

In studies of platelet lymphocyte ratios, we can see that the increase in these ratios indicates a poor prognosis of cancer, as well as showing predictive features for the need for intensive care in COVID-19 patients [29, 30]. In our study, it was concluded that the increase in PLR may be a candidate inflammatory marker. In addition, we found a weak but statistically significant correlation between the increase in PLR values and EDSS scores.

Unlike existing studies, we evaluated the SII value, which is accepted as a new inflammatory indicator combining information obtained from platelet, neutrophil and lymphocyte counts, in MS patients and control groups. Our results were found to be higher in MS patients than in healthy controls. In addition, SII values in patients with active lesions were also significantly higher than those without active lesions.

It should be kept in mind that conditions other than MS activity may also increase inflammatory indicators such as NLR, PLR, MLR and SII. Similar to previous studies conducted with these inflammatory indicators, patients with active infection, systemic inflammatory disease, or those receiving medical treatment that could affect the number of blood parameters were excluded from the current study. Therefore, white blood cell counts and CRP values in our study were also within normal limits.

Multiple sclerosis is a common autoimmune demyelinating disease of the central nervous system (CNS). Although the exact cause of MS is unknown, it is thought to have a multifactorial etiology, primarily environmental and genetic factors. While HLA variant HLA-DRB1*15:01, one of genetic factors, is kept responsible, environmental factors such as Epstein bar virus infection, low vitamin D level or smoking are also on the list [31]. There are two important hypotheses in the pathophysiology of MS [32]. The first is the inside-out hypothesis, which suggests that the autoimmune degenerative process initial starts in the CNS; however, there is insufficient evidence to support this hypothesis. The other is the outside-in hypothesis, which is commonly recognized, unlike the former hypothesis. In the outside-in hypothesis, it is assumed that autoreactive CD4+ T cells, which are divided into subgroups according to their pro- and anti-inflammatory cytokine profiles, are activated in the periphery and reach the CNS by crossing the blood‒brain barrier (BBB) [31, 33]. It has been proven by both experimental autoimmune encephalomyelitis (EAE) model and MS treatment studies that CD4+ T cells that migrate to the CNS are activated by antigen-presenting cells and direct leukocytes such as T and B cells and macrophages to initiate myelin damage [33, 34]. This inflammatory process triggers the degradation of myelin and the release of new CNS antigens called peripheral epitope spread [33]. The resulting persistent inflammation results in further damage to myelin and oligodendrocytes and axonal loss. While T and B cells, microglia, macrophages, complement and antibodies directly contribute to this observed damage, proinflammatory factors mostly generated by neutrophils, such as nitric oxide, matrix metalloproteinases (MMPs), tumor necrosis Factor a (TNF-α) and IL-1β, also play an indirect role in this damage [31]. The common point of these hypotheses is the triggering of the systemic inflammatory response in MS patients. In this respect, an increased incidence of other autoimmune diseases characterized by a systemic inflammatory response (such as Sjögren's syndrome and rheumatoid arthritis) in MS patients was observed [35, 36].

It has been shown through EAE models that neutrophils can act on the brain parenchyma by disrupting the BBB and increasing inflammation in MS. Neutrophils, which are short-lived cells, have been found to be highly expressed in MS patients at the onset of disease and relapse. In particular, neutrophils are thought to contribute to the pathological process by affecting the periphery rather than the CNS. In light of this information, myeloid cells such as neutrophils seem to be a target for future treatments, follow-up and monitoring [37]. Recent studies have reported that NLR, recognized as a new inflammatory biomarker, was higher in both MS and clinically isolated syndrome patients than in healthy individuals, which corroborates our study. This rate was also found to be associated with relapse, increased disability, and brain atrophy [13, 14, 34].

The radiological and histological indicator of MS disease is demyelinating plaques. Significant activation of microglia is present at the edge of active demyelinating lesions, while macrophages are at the lesion center, and it is unknown whether they are derived from a pool of activated microglia or from hematogenous monocytes. Similarly, a high number of microglia are present in the periplate and even in the distant, normal-appearing white matter [38]. MS plaques are typically characterized by BBB damage, which allows antigen-presenting cells such as B cells and myeloid cells to migrate through the BBB, leading to the differentiation of memory T cells into proinflammatory T helper lymphocytes [39].

Platelet abnormalities in MS patients were reported decades ago [40, 41]. New information supporting these studies is the detection of platelet-specific GPIIb (CD41) in brain tissues in human MS plaques and EAE models [42]. In addition, platelet activating factor (PAF) levels in CSF have been associated with both EAE and MS disease activity [38]. Interestingly, reduced PAF receptor levels led to reduced inflammation and demyelination in mice with EAE [39]. Recently, gangliosides GT1b and GQ1b, which are specifically recognized by platelets in the brain, and brain-specific glycolipids in the perivascular space area have been shown to trigger immune response cascades [41]. EAE attenuation in mice, especially in the active phase of the disease, when platelets are reduced both in number and function, clearly demonstrated the important contribution of platelets in the pathogenesis of EAE. Similarly, leukocyte migration to the inflamed CNS is limited by the reduction of platelets both in number and function [43]. However, there is a few study conducted on MS patients related to platelet count, which has been shown to be important in the pathophysiology of MS in the literature. In our study, PLR was found to be higher in the patient group than in the control group, supporting this view. In patient subgroups, a significant increase in PLR in radiologically active patients may have meaning as an inflammatory biomarker.

SII, a new indicator calculated by combining the information from all three of the platelet, neutrophil and lymphocyte counts, has been recently shown to be an independent risk factor in determining the severity of the disease and short-/long-term prognosis in acute cerebrovascular events [44, 46]. The SII was also found to be an independent new indicator for the development of respiratory failure in acute inflammatory demyelinating polyneuropathy [47]. Apart from this, SII levels were also investigated in Parkinson's disease, which is known to be a neurodegenerative disease, and it was established that SII value increases with increasing motor impairment, leading to negative effects on daily living activities [48]. In our study, SII levels elevated in MS patients with and without radiologically active lesions compared with healthy controls, SII levels were found to be significantly higher in patients with active lesions than in patients without active lesions such as NLR, MLR, and PLR. In addition, there was a positive correlation between disease duration and EDSS scores with PLR values. However, there was no significant relationship between MLR, NLR and SII measurements and disease duration in either group. Similarly, no statistically significant correlation was found between treatment status, number of lesions, and MLR, PLR, NLR, and SII measurements in the patient group with and without active lesions.

The NLR, MLR, PLR and SII, which we found to be higher in patients with active lesions, seem to be important in terms of indicating the inflammatory period of MS patients. In addition, according to the results of our study, it can be suggested that the presence of active lesions can be detected without using gadolinium with the cutoff value we determined for MLR, PLR, NLR and SII in patients with active lesions on MRI based on the results of our study. Among these rates, the SII value, including PLR, becomes prominent because it combines information from three blood parameters, unlike other inflammatory indicators obtained by the rate of two blood parameters, and it has high susceptibility and specificity.

It has recently been shown that gadolinium, which is the contrast agent for MRI that is frequently used in the monitorization of MS patients, can accumulate in many tissues, especially in the brain, and may have long-term toxic effects [49]. Therefore, SII values above 434.45 can be considered a noninvasive, inexpensive and easily accessible alternative with high susceptibility and sensitivity.

Our study has several limitations. First, our study was designed retrospectively. Therefore, only the leukocyte count and CRP level were evaluated among the inflammatory markers, and the levels of other inflammatory markers could not be measured. This did not allow us to make further comments on the highly complex pathophysiology of MS. Similarly, only patient sera were used in our study, and inflammatory indicators in CSF could not be evaluated. In addition, only patients with radiological activity in the presence of gadolinium were included in our study, and more sensitive studies could not be performed. While determination of attack frequency and disability is clinically important for follow-up and treatment monitoring in MS patients, radiological evaluation is another vital parameter. The inflammatory process is thought to continue in an exacerbated form in radiologically active patients. The high level of new inflammatory biomarkers in our study in patients with active lesions also lends support to this information. Patients benefit more from treatment in this phase of the disease rather than in the chronic neurodegenerative phase, and the disability rate of patients who receive effective treatment decreases in the following period. SII, an important inflammatory biomarker in MS pathophysiology that combines information from platelet, neutrophil and lymphocyte counts, which was found to be correlated with radiological activity in our study, can be considered an easily accessible option that does not require additional cost in the follow-up of MS patients and treatment monitoring in the inflammatory phase, during which MS medications are more effective. In addition, our results, which are a reflection of radiological activity, may be an alternative option for the use of gadolinium. Further studies in larger patient series and subgroups are required to support this information and make more interpretations.

## Data Availability

The datasets generated and/or analyzed during the current study are not publicly available. These data contain direct and indirect identifiers, but are available from the corresponding author upon reasonable request.
